# Characterization of Fatigue Properties of Fiber-Reinforced Polymer Composites Based on a Multiscale Approach

**DOI:** 10.3390/polym17020157

**Published:** 2025-01-09

**Authors:** Hyeonseok Han, Yuen Xia, Sung Kyu Ha

**Affiliations:** Department of Mechanical Engineering, Hanyang University, 222 Wangsimri-ro, Seongdong-gu, Seoul 04763, Republic of Korea; hhs4607@hanyang.ac.kr (H.H.); xiayuen@hanyang.ac.kr (Y.X.)

**Keywords:** characterization of fatigue properties, multiscale analysis, micromechanics, polymer matrix composites, temperature-dependent properties

## Abstract

This study presents a methodology for characterizing the constituent properties of composite materials by back-calculating from the laminate behavior under fatigue loading. Composite materials consist of fiber reinforcements and a polymer matrix, with the fatigue performance of the laminate governed by the interaction between these constituents. Due to the challenges in directly measuring the properties of individual fibers and the polymer matrix, a reverse-engineering approach was employed. Using the micro-mechanics of fatigue (MMFatigue), we predicted the laminate’s fatigue behavior based on assumed constituent properties and compared these predictions with experimental data from fatigue tests. The properties of the fiber and polymer matrix were iteratively adjusted to minimize the differences between predictions and experimental results, enabling accurate fatigue characterization. To ensure robustness, three laminate angles—0°, 30°, and 60°—were evaluated at three temperatures: low temperature (LT: −40 °C), room temperature (RT: 25 °C), and high temperature (HT: 85 °C). The error, defined as the fatigue life difference between the prediction and the experimental results, were obtained as 2.48% at LT, 7.18% at RT, and 1.25% at HT for a laminate angle of 45°. Finally, the applicability of the multiscale-based fatigue life prediction method was demonstrated through studies on laminates with various angles under tension–compression, and compression–compression cyclic loads, as well as composite pressure vessels under cyclic loading.

## 1. Introduction

Fiber-reinforced polymer composite materials have been steadily demanded across various industries due to their exceptional strength-to-weight ratio, corrosion resistance, and fatigue resistance. These properties make composites an alternative to traditional materials, like metals, particularly in applications that require weight reduction, and enhanced performance, such as long-term behavior. Indeed, aircraft wings, hydrogen storage pressure vessels, composites parts, and wind turbine blades increasingly rely on the composite materials [1]. The ability of the materials to withstand high loads while being significantly lighter than metals has been invaluable in improving fuel efficiency, reducing operational costs, and extending the service life [2].

Despite these advantages, the design of composites considering long-term behavior is limited due to their complex fatigue behavior. Unlike homogeneous materials, such as aluminum or steel, composites consist of fibers and a matrix, each possessing distinct mechanical properties. The interaction between these two constituents significantly affects the fatigue failure of laminated composites [3].

Fiber-reinforced composite laminates exhibit different mechanical properties, fatigue, and durability characteristics, and application scenarios depending on the type of fibers (carbon fiber, glass fiber, basalt fiber) and resin matrix (thermosetting and thermoplastic resins) are used [4,5,6]. The fatigue properties of different FRPs can vary significantly based on the characteristics of the fibers and resins as well as the manufacturing process. To understand these composite characteristics, applying a multiscale technique, which involves characterizing the fiber and resin separately, is essential.

The fatigue failure mechanisms of fiber-reinforced composites show distinct differences between unidirectional longitudinal (UDL) composites and biaxial (BX) laminates due to their structural configurations [7,8]. In UDL composites, fatigue loading induces cracks that progressively grow, leading to fiber failure or debonding between the fibers and the matrix. By contrast, BX laminates, with fibers arranged perpendicularly in two directions, experience fatigue failure primarily due to matrix cracking. These cracks cause stress concentrations within the matrix, eventually resulting in debonding between the fibers and the matrix. Such differences serve as important criteria for distinguishing the fatigue failure mechanisms of UDL and BX laminates.

Early theories on fatigue analysis in composites, developed by researchers like Hashin and Rotem [9] and Ellyin and El-Kadi [10], aimed to classify fiber and matrix failures based on laminate angles. These early theories laid the groundwork for modern multiscale analysis and helped deepen our understanding of the heterogeneous properties of composite materials. However, the anisotropic nature of composites made accurate fatigue life predictions difficult, requiring lots of tests with varying stress ratios and laminate angles.

Sayyidmousavi et al. [11] developed a multiscale fatigue analysis based on Aboudi’s method of cells [12]. In this model, fiber-direction stresses were considered indicative of fiber failure, while transverse and shear-direction stresses indicated matrix failure, creating a fatigue damage model. Fatigue life predictions were performed to laminate specimens at various angles (0°, 30°, 45°, 60°, 90°). In this study, tension and compression fatigue (R = 0, ∞) were investigated; however, the mean stress effect, which is necessary to account for various stress ratios, is not considered.

Brunbauer et al. [13] developed a fatigue model based on Puck’s criterion to distinguish between fiber and matrix fatigue failure, allowing for the prediction of fatigue life. Fatigue test results from three laminate angles (0°, ±45°, 90°) and two stress ratios (R = 0, R = −1) were used to analyze fatigue damage parameters. Additionally, the influence of stress ratios was examined using Haigh diagrams. In this study, each laminate angle was analyzed independently, treating them as having different material properties. This approach required a significant number of tests when considering various laminate angles.

Kumar et al. [14] used a modified Gerber model based on both creep and fatigue, with the Tsai–Hill model. This model was used to predict the lifespan of composites at various stress ratios (R = 0, 0.2, 0.4, 0.6) and laminate angles (15°, 30°, 45°, 60°). The finite element method (FEM) was used to analyze the stresses in the fiber and matrix under fatigue loads. Due to the limitations of the model, predicting the fatigue life under compressive loading is not considered.

Yun et al. [15] developed a fatigue analysis model based on a multi-level damage approach that considers sequential interface debonding using Eshelby’s tensor [16]. Damage parameters were obtained from stiffness degradation experiments conducted by Shokrieh and Lessard [17] under fatigue loads in longitudinal, transverse, and in-plane shear directions. The results aligned well with the fatigue test data, and the model was validated across various angles and laminates. The model for different stress ratios needs to be validated to effectively predict overall fatigue behaviors. Additionally, the progressive fatigue model made damage accumulation unequal across fatigue cycles, complicating rain-flow counting.

The research team led by Ha [18] developed a fatigue model, the micro-mechanics of fatigue (MMFatigue), based on the micro-mechanics of failure (MMF) [19]. Using a micromechanics model combined with the finite element method (FEM), they categorized the stresses acting on the fiber, matrix, and interface [20]. They also developed a matrix failure model by classifying the tensor stresses on each constituent into equivalent stress. By incorporating the mean stress effect in fatigue loading and employing a modified Goodman model that accounts for the different strengths in tension and compression, this model can be applied to various stress ratios. This approach was validated using various laminate angles (0°, 15°, 30°, 45°, 60°, 90°) of GFRP.

The recent fatigue analysis models are summarized in Table 1, outlining the key features of each approach. Most models accommodate different biaxial angles using a classical laminate theory (CLT) [21], which helps explain the behavior of composite layers subjected to loads at various orientations. The multiscale approach is widely adopted, as it is crucial to differentiate between the fiber and matrix components of the composite since their interaction plays a significant role in fatigue failure. A few models consider various stress ratios including tension–tension, tension–compression, and compression–compression fatigue loads, typically relying on a constant-life diagram (CLD) to manage these different loading conditions. Damage accumulation under sequential fatigue loading is simplified using Minor’s rule [22,23] and rain-flow counting. However, it is quite challenging to use this method in progressive fatigue models, where damage is not simplified with fatigue life. Therefore, the damage of composite based on fatigue life is still well recognized and used in the design of composite structures.

The multiscale fatigue analysis of fiber-reinforced composites requires accurate characterization of constituent material properties for reliable fatigue life prediction. However, directly measuring the properties of fibers and the matrix through separate experiments is challenging due to differences from actual manufactured composites, making accurate predictions through the multiscale approach difficult. This complexity arises from issues such as voids, residual stress, and fiber waviness caused by the manufacturing process, leading to changes in mechanical properties that are difficult to capture through standard experimental methods.

To address this challenge, this study adopts a back-engineering approach that considers the manufacturing process to determine the effective material properties of fibers and the matrix. This method enables the characterization of properties as they exist within the composite structure, accounting for process-induced effects. While past research has relied on experimental testing of laminated composites, no systematic method has been established for accurately characterizing individual constituent properties using multiscale fatigue analysis [11,13,14,15,18].

This study proposes a novel approach by integrating micromechanics-based fatigue analysis with back-engineering, providing a systematic method for determining temperature-dependent material properties. These properties are then applied to multiscale fatigue analysis, enabling accurate fatigue life predictions under varying fiber orientations and environmental conditions. Using the MMFatigue model, the laminate’s fatigue behavior is predicted based on constituent properties and compared with experimental data from fatigue tests. The properties of the fiber and polymer matrix are iteratively adjusted to minimize the difference between predicted and experimental results, enabling accurate characterization from three laminate orientations, i.e., 0°, 30°, and 60°, and evaluating their fatigue behavior at three different temperatures: −40 °C, 25 °C, and 85 °C. With this approach, the fatigue behavior of laminated polymer composites can be well characterized under fatigue loads and temperature-varying environments.

## 2. Methodology

**Figure 1:**
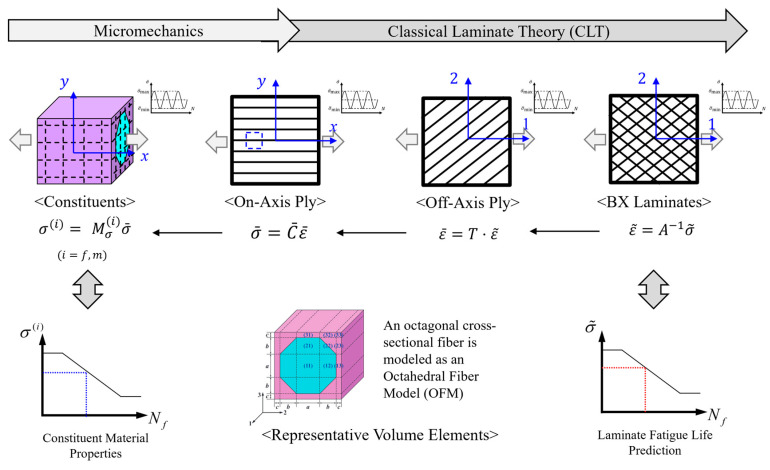
Flow chart of fatigue prediction of composite laminates using multiscale approach.

Physically, constituents of fiber and polymer matrix construct a ply, thus the properties of a ply are determined by the properties of constituents and their respective content ratios. The plies are then stacked and laminated with ply angles, forming a laminate. If the laminate consists of balanced angles, such as plus and negative angles, with respect to a reference axis, the laminate is called a biaxial (BX) laminate. Obviously, the mechanical response of the BX laminate under mechanical and environmental conditions is determined with the constituent properties. The current procedure for the fatigue analysis of fiber-reinforced composite laminates using a multiscale approach is illustrated in Figure 1.

For computation of the mechanical properties of the ply, a representative volume element (RVE) is used to represent a unit cell in a repeated array of fibers within the polymer matrix with the fiber volume fraction. With the RVE, the effective ply properties are obtained with proper repeated boundary conditions [20], and the stress concentration due to the presence of fiber within a ply can be also obtained from the RVE. However, the constituent properties are difficult to measure, and they can vary during the manufacturing process. To overcome this, the approach defines and determines key property factors essential for constituents using an iterative analysis until matching with laminate-based test data. This approach is defined as a reverse-engineering approach, which is described in Section 2.2.3. This process allows for the functional determination of temperature-dependent constituent properties, enabling fatigue life predictions for laminates across various temperatures. During the process, once the ply properties are obtained, the mechanical behavior of the BX laminates is determined from the ply properties and stacking ply angles, as well defined as in CLT [21].

### 2.1. Multiscale Approach for Fatigue Life Prediction

The micromechanics model used in this study is the octahedral fiber model (OFM) [24], where a circular fiber is modeled as an octagonal fiber, as depicted in Figure 1 The octagonal fiber, characterized by its eight-sided cross-sectional shape, provides an accurate representation of fiber yet enables an analytical approach in micromechanical modeling. With this micromechanical model, the effective ply properties are calculated and the stress amplification factor (SAF) is determined from the elastic modulus of the constituents and the fiber volume fraction (*V_f_*). Computational calculation of the complex behavior at the micro-level is made possible thanks to the OFM approach.

The effective stress–strain relationship of the unidirectional ply is represented as follows:

(1) $$\bar{\sigma }=\bar{C}\bar{\varepsilon },\;\bar{S}={\bar{C}}^{-1}$$

where $\bar{\varepsilon }$ is the strain tensor of the ply, $\bar{\sigma }$ is the stress tensor applied to the ply, $\bar{S}$ is the macro compliance of the unidirectional ply, composed of the fiber elastic moduli (*E_f_*_1_, *E_f_*_2_, *v_f_*_12_, *v_f_*_23_, *G_f_*_12_, *G_f_*_23_), the polymer matrix elastic moduli (*E_m_*, *v_m_*), and the fiber volume fraction (*V_f_*).

(2) $${\bar{E}}_{x}=\frac{1}{{\bar{S}}_{11}},{\bar{E}}_{y}=\frac{1}{{\bar{S}}_{22}},{\bar{v}}_{yx}=-\frac{{\bar{S}}_{21}}{{\bar{S}}_{11}},{\bar{v}}_{xy}={\bar{v}}_{yx}\frac{{\bar{E}}_{y}}{{\bar{E}}_{x}},{\bar{G}}_{xy}=\frac{1}{{\bar{S}}_{66}}$$

where ${\bar{E}}_{x}$, ${\bar{E}}_{y}$, ${\bar{v}}_{xy}$, and ${\bar{G}}_{xy}$ represent the ply effective properties, and *S_ij_* are the components of the compliance matrix.

The SAF represents the relationship between the micro stresses and macro ply stresses at each local points in the OFM [24].

(3) $$\sigma^{\left(i\right)}=\;M_{\sigma }^{\left(i\right)}\bar{\sigma }$$

(4) $${\left(\begin{array}{c} \sigma_{1}\\ \sigma_{2}\\ \sigma_{3}\\ \sigma_{4}=\tau_{23}\\ \sigma_{5}=\tau_{31}\\ \sigma_{6}=\tau_{12} \end{array}\right)}^{\left(i\right)}={\left[\begin{array}{cccccc} M_{11} & M_{12} & M_{13} & M_{14} & 0 & 0\\ M_{21} & M_{22} & M_{23} & M_{24} & 0 & 0\\ M_{31} & M_{32} & M_{33} & M_{34} & 0 & 0\\ M_{41} & M_{42} & M_{43} & M_{44} & 0 & 0\\ 0 & 0 & 0 & 0 & M_{55} & M_{56}\\ 0 & 0 & 0 & 0 & M_{65} & \alpha \cdot M_{66} \end{array}\right]}^{\left(i\right)}\left(\begin{array}{c} {\bar{\sigma }}_{1}\\ {\bar{\sigma }}_{2}\\ {\bar{\sigma }}_{3}\\ {\bar{\sigma }}_{4}={\bar{\tau }}_{23}\\ {\bar{\sigma }}_{5}={\bar{\tau }}_{31}\\ {\bar{\sigma }}_{6}={\bar{\tau }}_{12} \end{array}\right)$$

where $\sigma^{\left(i\right)}$ is the micro-stress tensor inside the fiber and matrix in the RVE, and $M_{\sigma }^{\left(i\right)}$ is the SAF matrix. $\bar{\sigma }$ are the on-axis stresses on the ply. The parameter α represents the shear stress reduction ratio applied to the shear term in the SAF matrix due to the nonlinear effect of the matrix with the interface.

**Figure A1:**
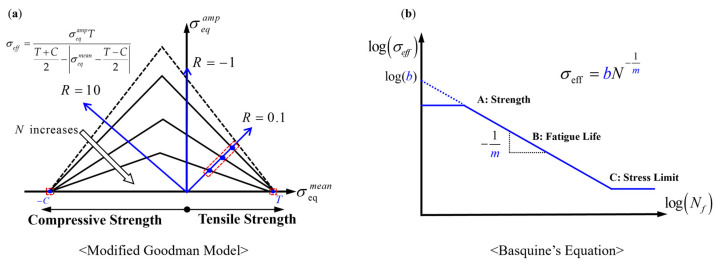
Graphical representation of: (**a**) modified Goodman model; (**b**) Basquin’s model.

In this approach, the MMFatigue theory was employed, as described in Appendix A. The fatigue behavior of composite materials under cyclic loads is analyzed by decomposing the applied fatigue loads into two main components: mean stress ($\sigma_{\mathrm{mean}}^{\left(i\right)}$) and amplitude stress ($\sigma_{\mathrm{amp}}^{\left(i\right)}$), as shown in Equation (3). These micro-stress components within the fiber and matrix are computed and then converted into equivalent stresses. The equivalent stresses are subsequently calculated to effective stresses using a modified Goodman model (Figure A1a).

The SN curve in this study follows Basquin’s model (Figure A1b) [25], and the equation is as follows:

(5) $$\sigma_{\mathrm{eff}}=b{N_{f}}^{-\frac{1}{m}}$$

where *N_f_* is the number of cycles to failure, *b* is the y-intercept of the logarithmic SN curve, and *m* is to its slope. *b* = *b_f_* and *m* = *m_f_*, when material is fiber, and *b* = *b_m_*, and *m* = *m_m_*, when material is matrix.

### 2.2. Characterization Method of Constituent Material Properties

#### 2.2.1. Parameters for Characterization Method

The parameters used in the overall multiscale approach, as described in Section 2.1, are presented in Table 2. To characterize the constituent properties for predicting test data, the parameters are classified into design variables (DVs), independent variables (IVs), and fixed values. DVs represent critical parameters optimized through comparison with laminate test data, while IVs reflect controllable environmental and specimen conditions in the tests. Fixed Values are parameters that do not significantly influence the test results and are thus excluded from the characterization process.

The elastic moduli, except for the longitudinal elastic modulus of fiber (*E_f_*_1_) and the elastic modulus of matrix (*E_m_*), are categorized as fixed values because their variations have little impact on the analysis outcomes. These fixed values are based on data from constituents of AS4/8552 used in the third world-wide failure exercise (WWFE-III) [26]. Additionally, the compressive strength of the fiber (*C_f_*) is assumed to be 70% of its tensile strength (*T_f_*), as fiber compression effects are not considered in this study. Furthermore, the shear stress reduction ratio (α), influenced by the matrix’s nonlinear behavior, is treated as a design variable (DV). The biaxial angle (θ) and fiber volume fraction (*V_f_*) are also considered independent variables (IVs). The biaxial angle can be controlled through the manufacturing process, while the fiber volume fraction can be measured experimentally.

These fixed parameters have a minimal impact on the analysis results. The proposed method is applicable to FRP composites with various combinations of carbon fibers and polymer matrices.

#### 2.2.2. Implicit Dependency of Laminates on Constituent Properties

The elastic modulus of laminates is implicitly influenced by constituent micro-level and macro-level factors, such as the elastic moduli of fiber and matrix, the fiber volume fraction, and the biaxial angle. These material properties are homogenized and appear as the elastic modulus of laminates.

The elastic modulus of UDL laminates (θ=0) is assumed to be typically dependent upon the fiber’s elastic modulus and volume fraction due to the much higher stiffness of the fiber. The elastic modulus of BX laminates is influenced by both fiber and matrix stiffness and fiber direction. Thus, the elastic moduli of UDL and BX laminates can be expressed in implicit functions as follows:

(6) $$\begin{array}{c} E_{\mathrm{UDL}}=f_{E_{\mathrm{UDL}}}\left(E_{f1},V_{f}\right)\\ E_{\mathrm{BX}}=f_{E_{\mathrm{BX}}}\left(E_{f1},E_{m},V_{f},\theta \right) \end{array}$$

where *E*_UDL_ is the elastic modulus of UDL laminates, and *E*_BX_ is the elastic modulus of BX laminates with a biaxial angle of θ. $f_{E_{\mathrm{UDL}}}$ and $f_{E_{\mathrm{BX}}}$ are implicit functions of the elastic modulus for UDL and BX laminates, respectively, and are based on constituent properties.

The strength of laminates is implicitly influenced by constituent micro-level failure factors, such as the strength of the fiber and matrix, and shear stress reduction ratio, including the parameters for Equation (6). These properties incorporate necessary properties for determining the failure of laminates based on failure criterion in the MMF.

The strength of UDL laminates is primarily dependent on the fiber, as most of the stress is applied along the fiber’s axial direction. By contrast, BX laminates are primarily matrix-dependent; as the angle increases in BX laminates, the primary stress shifts to the matrix. Thus, the strength of UDL and BX laminates can be expressed in implicit functions as follows:

(7) $$\begin{array}{c} X_{\mathrm{UDL}}=f_{X_{\mathrm{UDL}}}\left(E_{f1},E_{m},T_{f},V_{f}\right)\\ X_{\mathrm{BX}}=f_{X_{\mathrm{BX}}}\left(E_{f1},E_{m},T_{m},C_{m},\alpha ,V_{f},\theta \right) \end{array}$$

where *X*_UDL_ is the strength of UDL laminates, and *X*_BX_ is the strength of BX laminates with a biaxial angle of θ. $f_{X_{\mathrm{UDL}}}$ and $f_{X_{\mathrm{BX}}}$ are implicit functions of the strength for UDL and BX laminates, respectively, and are based on constituent properties.

The fatigue life of laminates is implicitly influenced by constituent micro-level factors of the SN curve, such as fatigue properties of the fiber and matrix, including the previous parameters for Equations (6) and (7). These material properties are for fatigue life prediction using the MMFatigue.

The fatigue life varies with load and stress ratio (${\tilde{\sigma }}_{max}$, *R*), and this must be accounted for in the calculation as IVs. For UDL laminates, fatigue life is fiber-dependent, whereas for BX laminates, it is matrix-dependent. Thus, the fatigue life of UDL and BX laminates can be expressed in implicit functions as follows:

(8) $$\begin{array}{c} N_{f,\mathrm{UDL}}=f_{N_{\mathrm{UDL}}}\left(E_{f1},E_{m},T_{f},C_{f},b_{f},m_{f},V_{f},{\tilde{\sigma }}_{max}\right)\\ N_{f,\mathrm{BX}}=f_{N_{\mathrm{BX}}}\left(E_{f1},E_{m},T_{m},C_{m},b_{m},m_{m},\alpha ,V_{f},\theta ,{\tilde{\sigma }}_{max}\right) \end{array}$$

where *N_f_*_,UDL_ is the fatigue life of UDL laminates, and *N_f_*_,BX_ is the fatigue life of BX laminates with a biaxial angle of θ. $f_{N_{f,\mathrm{UDL}}}$ and $f_{N_{f,\mathrm{BX}}}$ are implicit functions of the fatigue life for UDL and BX laminates, respectively, and are based on constituent properties.

#### 2.2.3. Determination of Constituent Properties via a Reverse-Engineering Approach

The assumed properties of the fiber and polymer matrix, as used in the model, are iteratively adjusted to minimize the difference between the predicted and experimental results. This process, defined as a reverse-engineering approach, refers to fine-tuning the key parameters required for prediction as outlined in Section 2.2.1, ensuring alignment with experimental results.

The errors are defined to assess the difference between the predicted and experimental results based on assumed constituent properties. In previous steps, constituent properties for elastic moduli, strength, and fatigue properties were used to predict the laminates behavior based on each constituent parameter. To accurately characterize these constituent properties, the trust-region algorithm was implemented to efficiently handle boundary constraints and to ensure that solutions remain within feasible regions throughout the characterization process. This algorithm’s ability to maintain solutions within practical limits makes it ideal for accurate characterization of material properties. The equation for the minimize error function is as follows:

(9) $$\arg\;\underset{x}{\min}\;e\left(x\right)=\sum_{i=1}^{m}\left|\frac{f^{\prime }-f\left(x\right)}{f^{\prime }}\right|\;\mathrm{subject}\;\mathrm{to}:\;x^{l}\leq x\leq x^{u}$$

where **x** are variables of constituent material properties, the function *f*(**x**) is defined as in Equations (6)–(8), and *f*′ is test data. **x***^l^*, **x***^u^* are the lower and upper bounds of constituent variables, respectively, to ensure the constituent properties are within practical value ranges.

**Figure 2:**
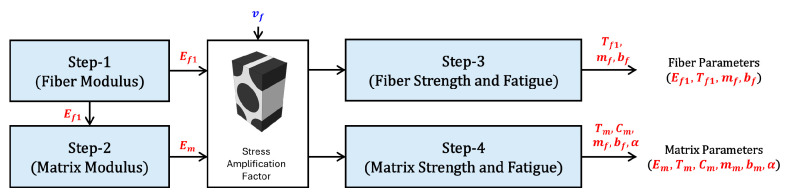
Characterization sequence for determination of constituent material properties.

To address the overlapping constituent properties, described as DVs, within each predicted laminate behavior, this process is divided into four sequential steps, as illustrated in Figure 2 and Table 3. Each step focuses on characterizing a specific DV by comparing predicted parameters with experimental data, then passing the characterized parameters to the next step.

The process begins with Step 1, where the elastic modulus of the fiber is characterized by comparing predicted values with the experimental results for the elastic modulus of UDL laminates. In Step 2, the elastic modulus of the matrix is characterized by comparing predictions with the experimental results for the elastic modulus of BX laminates. Then, to perform failure analysis of the constituents using a multiscale approach, a micromechanics model is constructed using the elastic modulus of the constituents obtained in Step 1 and Step 2. Step 3 and Step 4 focus on determining the strength and fatigue properties of the fiber and matrix, respectively. Since strength and fatigue properties are interdependent, the characterization process employs a combined error function to minimize discrepancies. Specifically, the strength and fatigue properties of the fiber are characterized based on the strength and fatigue life of UDL laminates, while those of the matrix are based on BX laminates.

Through these processes, the constituent material properties of the fiber and matrix for multiscale fatigue analysis can be separately determined. This approach ensures that both fiber and matrix properties are accurately characterized, providing a solid foundation for reliable multiscale fatigue analysis.

### 2.3. Temperature-Dependent Constituent Properties

Composite materials can be sensitive to temperature variations due to the polymer matrix. For example, polymers tend to lose stiffness and strength at high temperatures, whereas polymers may exhibit increased stiffness and strength at low temperatures [27,28]. The fatigue behavior of composites would show a similar pattern, but the modeling can be complicated. In this approach, the temperature dependent of the polymer resin, as listed in Table 2, is assumed to vary quadratically over the temperature, as shown Equation (10).

(10) $$p=a_{p}(Tr-25{)}^{2}+b_{p}(Tr-25)+c_{p}$$

where *a_p_*, *b_p_*, and *c_p_* represent polynomial constants for each constituent property (*p*). This simplifies the multiscale analysis of temperature-dependent composites, allowing the fatigue analysis to be performed over a range of temperatures.

## 3. Static and Fatigue Tests for a Reverse-Engineering Approach

This section describes the experimental procedure used to determine the fatigue properties of the constituent materials. The testing angles for the composite laminate experiments were selected to derive the material properties of the fiber and polymer matrix. The UDL was chosen to assess the fiber’s longitudinal properties, as this angle best captures the directional characteristics of the fiber. For polymer matrix properties, BX laminates at angles of 30° and 60° were utilized to consider both tensional and shear interaction between the polymer matrix and the fibers within a ply. In addition, both BX specimens can be prepared from the same panels.

Furthermore, the tests were conducted at three different temperature conditions, including low temperature (LT: −40 °C), room temperature (RT: 25 °C), and high temperature (HT: 85 °C), to characterize the constituent properties under different temperature conditions as needed in the model.

### 3.1. Manufacturing Process of Laminate Specimens

The carbon fiber used in this study was Toray’s T700, widely used in composite structures. The polymer matrix was Recyclamine^®^ EPOTEC YDL 5569 and THR 9351 from Aditya Birla, recently developed for recyclable composite applications, such as wind turbine blades and hydrogen gas storage tanks. This matrix system not only offers excellent mechanical properties but allows for chemical recycling, enabling the recovery of both fibers and resin. This makes it ideal for sustainable applications by reducing waste and promoting material reuse.

**Figure 3:**
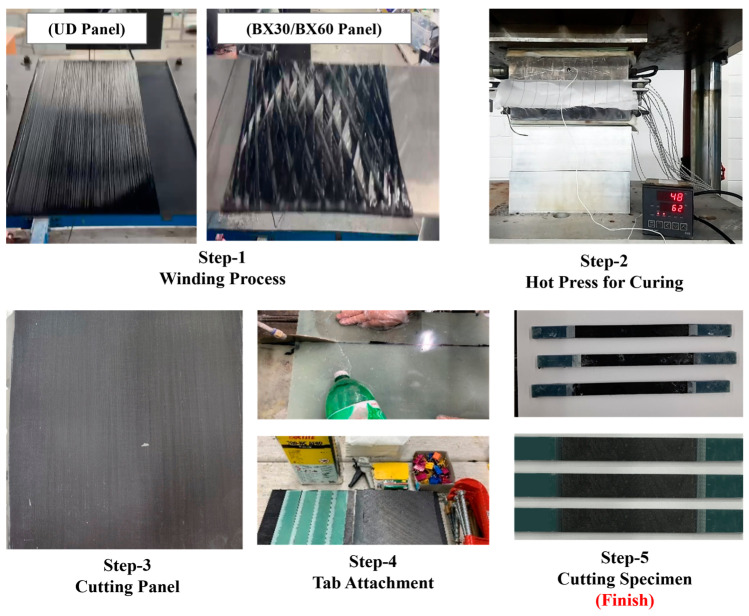
Manufacturing processes of specimens using a filament winding machine.

The panels were fabricated via a filament winding process at specific angles as illustrated in Figure 3. The winding angles were [0°]*_s_*, [+30°/−30°]*_s_*, and [+60°/−60°]*_s_*, UDL, BX30, and BX60 laminates, respectively, as shown in Table 4. After winding, the panels were cured under a constant pressure of 0.65 MPa at 80 °C for 4 h, followed by an additional 4 h at 120 °C. Specimens were prepared by attaching tabs with acrylic-based Loctite 401 adhesive and cutting them to the specified angles for testing according to ASTM-D3039 standards [29].

The fiber volume fraction of the manufactured panels was determined by measuring the composite’s density using Archimedes’ principle, followed by a calculation based on the rule of mixtures [30]. The calculation is performed using the following equation:

(11) $$V_{f}=\frac{\rho_{c}-\rho_{m}}{\rho_{f}-\rho_{m}}$$

where $\rho_{c}$ represents the density of the composite, measured using Archimedes’ principle by immersing the samples in water. The densities of fiber ($\rho_{f}$) and matrix ($\rho_{m}$) were referenced from the manufacturer’s technical data sheets (TDS) as 1.8 g/cm^3^ and 1.17 g/cm^3^, respectively [31,32].

A fiber volume fraction of 60% was measured for the UDL laminates, while the BX30 and BX60 laminates showed a fiber volume fraction of 52%. This difference arises from the stacking methods used. In UDL laminates, fibers are stacked in the same direction with minimal gaps, maximizing fiber volume. By contrast, BX laminates are stacked with alternating layers, creating more gaps and reducing the fiber volume fraction.

### 3.2. Static Test of Laminates

Mechanical tests were performed to measure the static properties of UDL, BX30, and BX60 laminates following ASTM-D3039 standards at three different temperatures: room temperature (RT: 25 °C), high temperature (HT: 85 °C), and low temperature (LT: −40 °C), with a strain rate as 1 mm/min.

The elastic moduli of the laminates were measured using strain gages while the elastic moduli at HT and LT were estimated based on homogenized properties calculated using the OFM and CLT. The longitudinal elastic modulus of the carbon fiber (*E_f_*_1_) was assumed to remain constant across all temperatures. Based on the study by Deng et al. [33], the polymer matrix elastic modulus (*E_m_*) at HT was assumed to be 74% of the RT value, while at LT, it was assumed to be 130% of the RT value. Using these temperature-dependent constituent properties, the estimated elastic moduli of laminates were calculated and applied to determine the constituent material properties.

**Figure 4:**
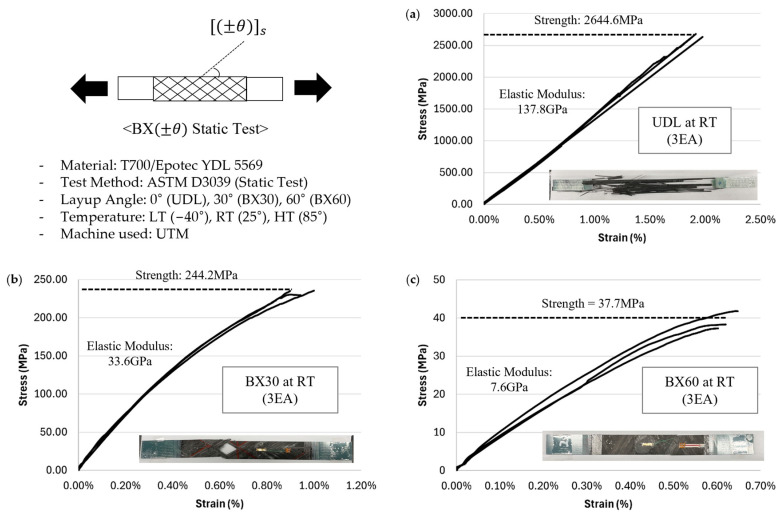
Stress–strain curve at RT: (**a**) UDL; (**b**) BX30; (**c**) BX60.

The elastic modulus of UDL, BX30, and BX60 reached 137.8 GPa, 33.6 GPa, and 7.6 GPa, respectively, while their tensile strengths reached 2644.6 MPa, 244.2 MPa, and 37.7 MPa. According to the TDS of Toray’s T700 [31], the elastic modulus and tensile strength of UDL laminates are 135 GPa and 2550 MPa, respectively, with an error of 2.07% in elastic modulus and 3.71% in tensile strength. Despite differences in the polymer matrix, the test data for UDL laminates are very similar to the reference data, supporting the reliability of the results. The static test results for UDL, BX30, and BX60 under RT conditions are shown in Figure 4, while the summary of averaged static test results under LT, RT, and HT conditions is presented in Table 5.

### 3.3. Fatigue Test of Laminates

**Figure 5:**
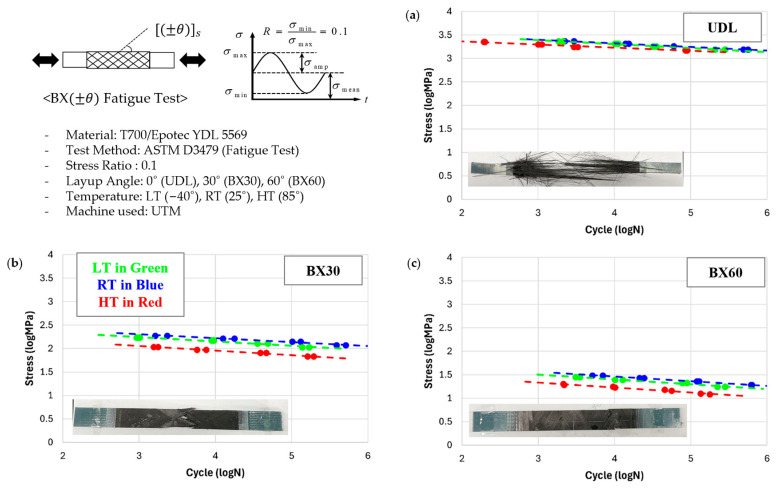
Measured SN curve at RT (in blue), HT (in red), and LT (in green): (**a**) UDL; (**b**) BX30; (**c**) BX60.

Fatigue tests were conducted on the same laminates: UDL, BX30, and BX60 following ASTM-D3479 standards [34] at room temperature (RT: 25 °C), high temperature (HT: 85 °C), and low temperature (LT: −40 °C). Each laminate was tested at four different load levels, i.e., 50%, 60%, 70%, and 80% of the ultimate strength of each laminate; the SN curves of the fatigue tests are shown in Figure 5. The stress levels were based on the ultimate strength rather than the yield strength because defining the yield strength in composites is challenging due to the nonlinear behavior caused by the polymer matrix.

In the experimental results, the strength and fatigue life were highest in the order of UDL, BX30, and BX60. This can be attributed to the increased stress on the high-strength carbon fiber as the biaxial angle decreases, which reduces the load applied to the relatively weaker polymer matrix, leading to higher strength and fatigue life.

In the fractured specimens of UDL laminates, the fiber breakage was accompanied by the separation of the polymer matrix. This observation suggests that, as the UDL laminate undergoes tensile loading, the fibers, which possess a higher elastic modulus, bear most of the load, leading to fiber failure. Subsequently, cracks form in the polymer matrix, resulting in fiber debonding. By contrast, the failure in BX laminates appears to originate from matrix cracking, which propagates and leads to the debonding of the fibers from the matrix. These observed failure mechanisms align well with the findings presented by Talreja [8].

Under different temperature conditions, strength and fatigue life were measured to be highest at RT, followed by LT and HT. Although RT and LT showed minimal differences, with less than a 15% variation across all laminates, a significant reduction was observed at HT, where reductions of more than 35% were seen in tests on BX laminates.

The slight decrease in strength at LT, which contrasts with typical composite behavior where strength tends to increase at low temperatures, can be attributed to the increased brittleness of the polymer matrix at lower temperatures. This brittleness makes the polymer matrix more susceptible to interface separation and failure [27]. During the filament winding process, voids can form at the interface, leading to separation and reducing composite strength and fatigue life compared to RT.

At HT, polymer degradation further reduces the strength and fatigue life of the composite. Polymers generally degrade progressively until reaching their glass transition temperature (Tg), beyond which strength reduction accelerates [28]. The HT condition of 85 °C in this experiment was sufficient to initiate degradation. However, in UDL laminates, carbon fibers bear a significant portion of the load, resulting in less reduction in strength and fatigue life compared to BX30 and BX60 under HT conditions.

## 4. Determination of Constituent Material Properties

The constituent material properties were determined, as described Section 2, using the experimental data of UDL, BX30, and BX60, as presented in Section 3. The calculated constituent properties with temperature coefficients are summarized in Table 6. Note that these were determined to best fit the MMFatigue theory, and the experimental data obtained from the winding manufacturing process.

### 4.1. Back Engineering Determination of Fiber Properties

The carbon fiber used in this study is Toray’s T700, which is often used in filament winding and prepreg applications. The elastic moduli, tensile strength, and the parameters for the SN curves with the temperature coefficients were determined, as shown in Table 6. At RT, the elastic modulus of the carbon fiber (*E_f_*_1_) was determined to be 227.37 GPa, aligning very well with Toray’s TDS [31] value of 230 GPa, with only a 1.1% discrepancy. However, the tensile strength (*T_f_*) at RT was determined to be 4367 MPa, which is 10.9% lower than the TDS value of 4900 MPa. It may be mainly due to micro-defects introduced during the manufacturing process. On the other hand, the elastic modulus is relatively insensitive to such micro-defects.

The tensile strength of the fiber (*T_f_*) was measured as 4367.8 MPa at RT, 4251.5 MPa at LT, and 4104.6 MPa at HT. This corresponds to a maximum decrease of approximately 5.3% at LT and 6.0% at HT compared to RT. These results quantitatively demonstrate that temperature has a moderate impact on the tensile strength of the fiber.

However, the slope of the fatigue SN curve of the fiber decreases, with a slope parameter (*m_f_*) of 7.66 at RT and 6.77 at LT. As reported by Cormier et al. [35], the slope of the SN curve for UDL may decrease at LT compared to RT. At HT, the slope became gentler, with a slope parameter of 8.05. Nevertheless, the overall fatigue performance at HT remained lower than at RT due to a significant reduction in strength.

### 4.2. Back Engineering Determination of Matrix Properties

The polymer matrix used in this study is Recyclamine^®^, an epoxy-based matrix. According to the TDS [32], the elastic modulus of the polymer matrix (*E_f_*) is 2.8 GPa, which is close to the back-calculated value of 3.11 GPa, showing a relative error of approximately 9.5%. This indicates reasonable agreement between the measured and reported values. The TDS-reported tensile strength (*T_m_*) of the polymer matrix is 72.5 MPa, while the back-calculated value is 47.7 MPa, reflecting a relative error of 34.2%, indicating a lower strength. This reduction is likely due to voids and defects formed during the manufacturing process, reflecting the polymer’s actual properties under manufacturing conditions. While some material properties, such as elastic modulus and tensile strength, are provided by TDS at RT, properties at HT and LT are not available in TDS.

In this study, UDL, BX30, and BX60 composites showed a decrease in strength at both LT and HT, largely due to the reduction in the polymer matrix’s strength under these conditions. The tensile strength and compressive strength of the polymer matrix (*T_m_*, *C_m_*) were highest at RT (47.7 MPa, 81.0 MPa) but dropped at HT (25.0 MPa, 35.1 MPa) and LT (37.6 MPa, 58.5 MPa). At LT, the polymer matrix became more brittle, making it prone to cracking, while HT reduced its strength due to thermal degradation. Similarly, the slope parameter of the SN curve for the polymer matrix (*m_m_*) was 4.9 at HT, 5.9 at LT, and 6.7 at RT. This trend underscores the polymer matrix’s susceptibility to temperature, being most vulnerable at HT and prone to failure because of brittleness at LT.

## 5. Verification Tests

In this section, as a verification of our approach, we predict the fatigue life of BX45 using material properties obtained from the UDL, BX30, and BX60 laminates, outlined in Section 3. The BX45 was fabricated using the same methodology as BX30 and BX60, and its strength and SN curves were measured to validate the accuracy of the predictions.

**Figure 6:**
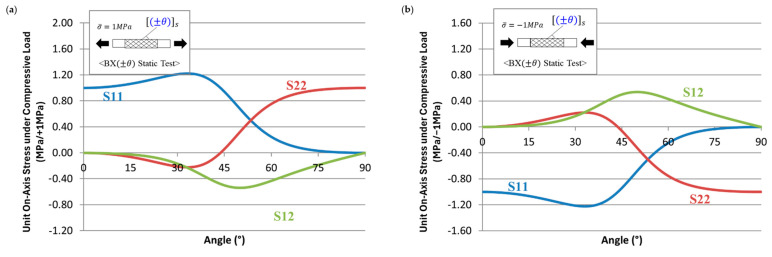
On-axis stress distribution across biaxial angle of laminates: (**a**) under tensile loading; (**b**) under compressive loading.

To better understand the variation of static strength, stiffness, and the fatigue SN curves along with the fiber angles, the on-axis stresses are calculated and plotted in Figure 6. For the BX30, BX45, and BX60 laminates, matrix failure played a significant role in fatigue performance. In this study, the compressive strength of the polymer matrix was determined that 1.7 times higher than the tensile strength. Based on Figure 6a, the calculation results of on-axis stresses across the biaxial angle of laminates using CLT [21], the transverse stress (*S*_22_) component in the on-axis stress distribution was observed to be compressive for BX30 under tensile loading, while for BX45 and BX60, it was tensile. Under compressive loading, as shown in Figure 6b, the transverse stress exhibited the opposite trend: BX30 experienced tensile loading, while BX45 and BX60 experienced compressive loading. Consequently, BX30 showed higher strength under compressive loading, while BX45 and BX60 exhibited lower strength. This suggests that the effect of the buckling mode, a primary failure mechanism in compressive failure, decreases as the biaxial angle increases.

**Figure 7:**
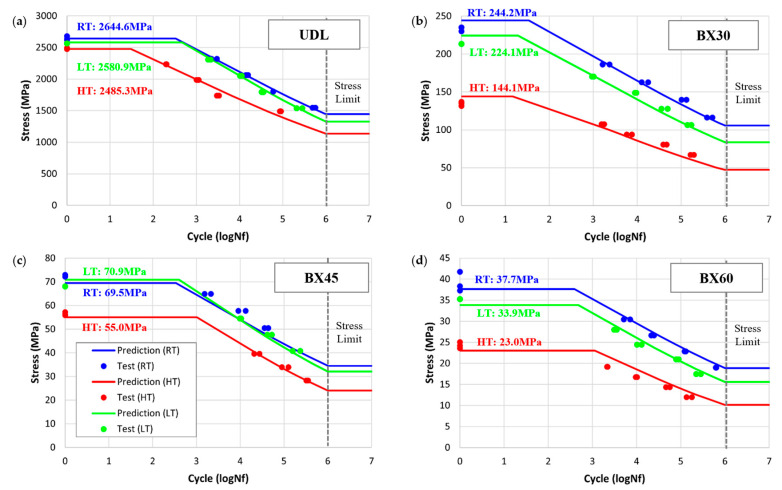
Predicted SN curve in RT (in blue), HT (in red), and LT (in green): (**a**) UDL; (**b**) BX30; (**c**) BX45; (**d**) BX60.

Figure 7 highlights the consistency of the predicted SN curves for BX45 compared with the experimental data for UDL, BX30, and BX60 under various conditions. The predicted SN curve was analyzed by dividing it into three parts—strength, fatigue life curve, and limit stress—according to Figure A1b in Appendix A. The fatigue life curve (line) was compared to individual fatigue test values (points), and the limit stress was determined for a fatigue life of 10^6^ (logNf = 6).

The error rates for each dataset were calculated using Equation (9), demonstrating the accuracy of the predictions. UDL primarily served to characterize the fatigue properties of fibers, with average error rates of 1.60%, 1.44%, and 4.57% under LT, RT, and HT conditions, respectively. BX30 and BX60 focused on characterizing the fatigue properties of the matrix, showing average error rates of 3.45%, 2.65%, and 7.69% under LT, RT, and HT conditions, respectively. BX45 was utilized as a validation dataset for predicting fatigue life at various angles, with error rates of 2.48%, 7.18%, and 1.25% under LT, RT, and HT conditions, respectively.

Additionally, the SN curves under LT conditions have minimal impact on the overall results compared to HT conditions, where higher temperatures (RT–LT = 65 °C; HT–RT = 60 °C) accelerate material degradation. This indicates that the resin properties under LT conditions are more stable and less sensitive to degradation compared to HT conditions.

**Figure 8:**
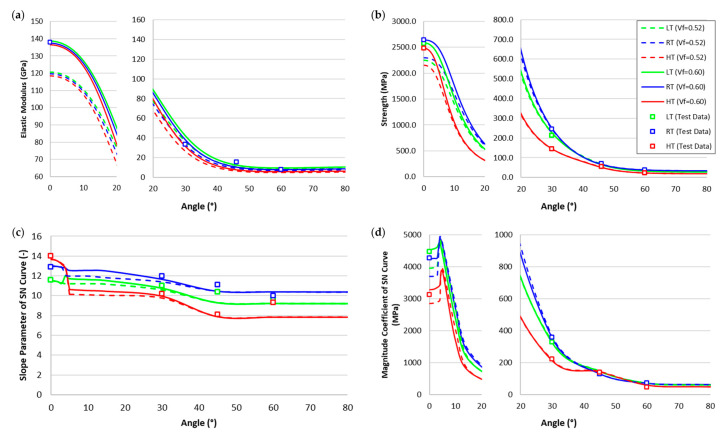
Prediction results of BX laminates under 0–80° and test data at RT (in blue), HT (in red), and LT (in green): (**a**) elastic modulus; (**b**) strength; (**c**) slope parameter of the SN curve; (**d**) magnitude coefficient of the SN curve.

The measured static elastic modulus and strengths of BX laminates, including the UD, were also compared with the predicted values for the angles from zero to 80 degrees, as shown in Figure 8a,b. Two different fiber volume fractions were considered in this study from 52% to 60%. Note that we achieved the fiber volume fraction of 52% for BX and 60% for the UDL laminates. Both strengths and stiffness are well predicted. It was observed that volume fraction influenced both the elastic modulus and strength predictions, especially between 0 and 20 degrees.

The measured SN curve’s magnitude coefficient (intercept) and slope parameter of BX laminates, including the UDL, were also compared with the predicted values for the angles from zero to 80 degrees, as shown in Figure 8c,d. The predictions were based on curve fitting using data at 80%, 70%, 60%, and 50% of the strength. Overall, UDL, BX30, BX45, and BX60 exhibited good agreement with experimental data, except for slight deviations in the SN slope of BX60 under HT and BX45 under LT. Although BX60 was included in the material property determination process, the polymer matrix’s nonlinear behavior at elevated temperatures resulted in deviations. In the case of BX45, which was not used in the property determination, predictions were made based on the behavior of BX30 and BX60. At low temperatures, the polymer matrix exhibited increased brittleness, leading to discrepancies with the original polymer prediction model. The material behavior predictions were based on the MMFatigue model [18], which relies on shear strain energy, suggesting that changes in shear behavior under brittle conditions caused slight deviations.

Discontinuities in the predicted data were shown in fiber and polymer matrix failure mode transition. Typically, at 0°, fiber dominates the behavior, while its influence diminishes as the angle increases. Transitions in failure modes were predicted around 4° under RT and LT conditions and around 5° under HT conditions. In fatigue tests, the fiber volume fraction had a significant influence, especially at near 0° angles, although the SN curve’s slope was largely unaffected by fiber failure.

The predictive analysis, validated through the verification process, demonstrated well agreement with experimental data across various conditions and layup angles, confirming the robustness of the proposed characterization and prediction methods. Although a divergence between fiber and matrix failure was observed around 4° to 5°, this had minimal impact on the overall assessment of laminate failure across different configurations. This study verified that the proposed characterization technique for the fatigue properties of composite materials is applicable to a range of layup angles and temperature conditions, confirming its potential for use in the fatigue design of composite structures.

## 6. Parametric Study

### 6.1. SN Curve of BX Laminate at RT for R = 0.1, R = 10, and R = −1

**Figure 9:**
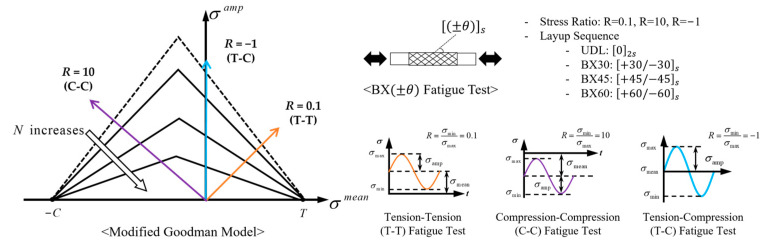
Fatigue analysis using a modified Goodman model for various stress ratios.

Parametric study was performed to predict fatigue analysis results of various stress ratios using a modified Goodman model, and specifically determined material properties by fatigue test under tension–tension fatigue tests (T-T), as shown in Figure 9. The analysis covers various laminate configurations, including UDL, BX30, BX45, and BX60. Here, the stress ratio is defined as the minimum load divided by the maximum load, and the analysis incorporates common stress ratios used in composite fatigue testing: *R* = 0.1, 10, and −1. Specifically, *R* = 0.1 corresponds to T-T fatigue tests, *R* = 10 to compression–compression fatigue tests (C-C), and *R* = −1 to tension–compression fatigue tests (T-C).

**Figure 10:**
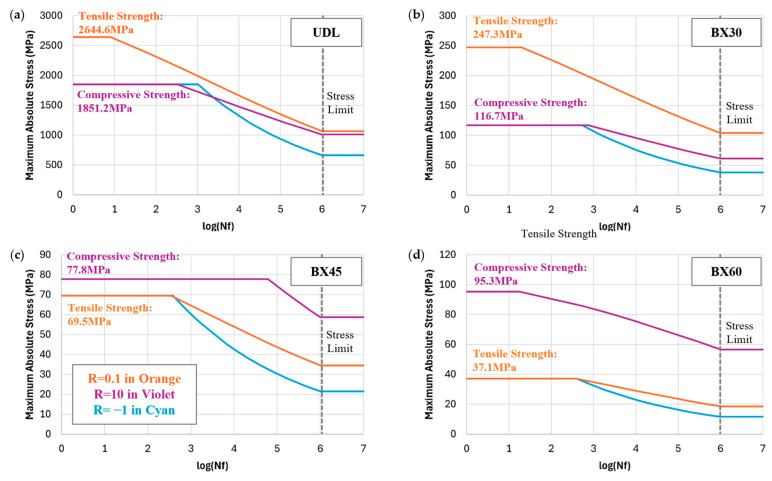
Prediction results of fatigue analysis under R = 0.1 (in orange), R = 10 (in violet), and R = −1 (in cyan): (**a**) UDL; (**b**) BX30; (**c**) BX45; (**d**) BX60.

Generally, in composite materials, the slope of the SN curve in C-C mode is less steep than in T-T mode, while it decreases sharply in T-C mode. This is because crack propagation is suppressed in C-C mode, whereas it is accelerated in T-T mode, resulting in higher fatigue resistance under C-C loading. However, for UDL laminates, the fibers are prone to buckling under high C-C fatigue loads, leading to microstructural failure before the material reaches ultimate failure [36]. This characteristic was incorporated into the modified Goodman model used for UDL, BX30, BX45, and BX60. The predicted SN curves of T-T, T-C, and C-C for UDL, BX30, BX45, and BX60 are shown in Figure 10. These SN curves were analyzed by dividing it into three parts—strength, fatigue life curve, and limit stress—according to Figure A1b in Appendix A.

For UDL laminates, fiber failure dominates, and the compressive longitudinal fiber strength was assumed to be 70% of the tensile longitudinal fiber strength. Thus, the compressive strength of UDL was estimated to be 1851.2 MPa, while the tensile strength was 2644.6 MPa. As a result, the compressive strength was predicted to be 70% of the tensile strength. Additionally, while the slope of the SN curve in T-C mode decreased sharply, it showed a gradual decline in C-C mode.

For BX laminates, polymer matrix failure is the primary cause, with the tensile strengths of BX30, BX45, and BX60 predicted to be 247.3 MPa, 69.5 MPa, and 37.1 MPa, respectively. These values are based on actual experimental data and were used to determine the strength of the polymer matrix. The compressive strengths for BX30, BX45, and BX60 were predicted to be 116.7 MPa, 77.8 MPa, and 95.3 MPa, respectively, with estimates derived from the tensile strength experimental values. For BX30, the tensile strength was predicted to be higher than the compressive strength, whereas for BX45 and BX60, the compressive strength was predicted to be higher than the tensile strength, as shown in Figure 6.

Regarding fatigue life, in T-C mode, the combination of tension and compression accelerates fatigue in the polymer, resulting in a sharp decline in fatigue life. By contrast, in C-C mode, the predicted fatigue life is higher than in T-C mode. This is because, due to the nature of polymers, fatigue crack propagation is suppressed under compressive conditions [36].

In this study, although compressive static and fatigue tests were not directly conducted, the derived material properties and the modified CLD model allowed for predicting fatigue behavior across various stress ratios. As a result, the analysis method of the modified Goodman model in the MMFatigue model has been proven effective for analyzing a range of stress ratios in composite laminates. This model enables more comprehensive fatigue analysis of fiber-reinforced composites, especially when experimental data are limited. However, for reliable fatigue predictions across various stress ratios, a CLD model based on extensive experimental data, such as a piece-wise linear constant life diagram [37], is necessary.

### 6.2. SN Curves of Double–Double Laminates Under Multiaxial Pressure Loads

**Figure 11:**
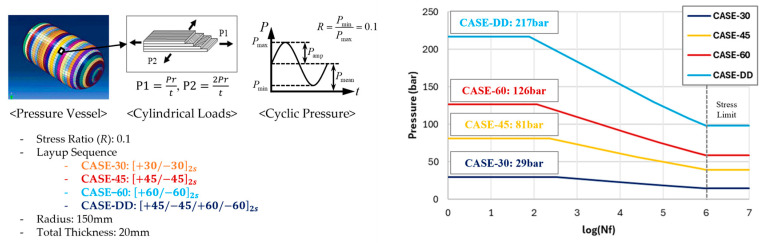
Prediction results of the SN curve for pressure vessel under multiaxial pressure loads.

This section demonstrates the application of the current multiscale-based fatigue life prediction of composite pressure vessels under cyclic loads. Pressure vessels are typically wound with multiple biaxial angle laminates. Figure 11 shows the comparison cases of three biaxial laminates and one double biaxial laminate subjected to the multiaxial loading resulting from internal pressure, which was applied with a stress ratio of 0.1. The predicted SN curve was analyzed by dividing it into three parts—strength, fatigue life curve, and limit stress—according to Figure A1b in Appendix A.

The layup CASE-30 means the biaxial laminate of [+30/−30]_2*s*_, the CASE-45 [+45/−45]_2*s*_, CASE-60 [+60/−60]_2*s*_, and CASE-DD, [+45/−45/+60/−60]_2*s*_, which is a double–double laminate, recently proposed by Steve Tsai [38].

The burst pressure for CASE-30 was predicted as 29 bar, CASE-45 as 81 bar, and CASE-60 as 126 bar, whereas CASE-DD exhibited a significantly higher burst pressure of 217 bar compared to the other cases. The increased burst pressure in CASE-DD can be attributed to the balanced laminate structure achieved through the double–double configuration, which are well demonstrated in this study. The entire SN curves are reasonably well obtained as well as the burst pressures. Without the current method, the tests would have taken months to perform the entire fatigue tests.

## 7. Summary

This study investigated a multiscale approach for characterizing fiber-reinforced polymer composites, focusing on material property determination and fatigue life prediction. The following conclusions were drawn based on the experimental and analytical findings:

- A reverse-engineering method was developed to determine the material properties of fiber-reinforced polymer composites.
- Static and fatigue tests were conducted on UDL and BX laminates at angles of 0°, 30°, and 60°, and results were validated using 45° BX laminates, confirming that fatigue life could be predicted with tests at only three specific angles.
- The elastic modulus values obtained were consistent with known data, while lower strength values were attributed to micro-defects introduced during the manufacturing process.
- Temperature changes significantly affected composite performance. At LT, strength and fatigue life decreased, while at HT, strength was reduced, and the SN curve became more gradual. The temperature-dependent constituent properties were functionally represented, enabling analysis across various temperature conditions.

Ultimately, this study succeeded in deriving temperature-dependent constituent properties, including the effects of manufacturing defects, enabling their application in the multiscale approach.

However, further validation of the current MMFatigue is necessary across a wider range of stress ratios. Currently, the model uses CLD based on a stress ratio of R = 0.1, which regresses tensile and compressive strength to predict fatigue life. As shown in the results of Section 6.1, the limitations of the current CLD approach indicate that it alone is insufficient for predicting fatigue life across various stress ratios. Future work should aim to verify this approach using other fatigue models, such as a piecewise-linear approach, to extend its applicability to a broader range of stress ratios.

## Figures and Tables

**Table 1:** Comparison of recent fatigue analysis models for polymer composites.

Model	Sayyidmousavi [11]	Brunbauer [13]	Kumar [14]	Yun [15]	Ha [18]
Issue date	July/2015	October/2015	September/2023	June/2024	August/2011
Various biaxial angle	YES	NO	YES	YES	YES
Multiscale approach	YES	NO	YES	YES	YES
Various stress ratio	NO	YES	YES	NO	YES
Rain-flow counting	YES	YES	YES	NO	YES

**Table 2:** Parameter list for multiscale fatigue analysis.

Group	Symbols	Definition	Parameters
Elastic moduli of fiber	*E_f_*_1_	Longitudinal elastic modulus of fiber	DV
	*E_f_*_2_	Transverse elastic modulus of fiber	15 GPa ^1^
	*G_f_*_12_	In-plane shear modulus of fiber	15 GPa ^1^
	*G_f_*_23_	Out-of-plane shear modulus of fiber	7 GPa ^1^
	*v_f_*_12_	In-plane Poisson’s ratio of fiber	0.2 ^1^
	*v_f_*_23_	Out-of-plane Poisson’s ratio of fiber	0.2 ^1^
Strength of fiber	*T_f_*	Tensile strength of fiber	DV
	*C_f_*	Compressive strength of fiber	70% of *T_f_* ^2^
Fatigue properties of fiber	*b_f_*	Slope parameter of SN curve of fiber	DV
	*m_f_*	Magnitude coefficient of SN curve of fiber	DV
Elastic moduli of matrix	*E_m_*	Elastic modulus of matrix	DV
	*v_m_*	Poisson’s ratio of matrix	0.35 ^2^
Strength of matrix	*T_m_*	Tensile strength of matrix	DV
	*C_m_*	Compressive strength of matrix	DV
Fatigue properties of matrix	*b_m_*	Slope parameter of SN curve of matrix	DV
	*m_m_*	Magnitude coefficient of SN curve of matrix	DV
Nonlinearity of matrix	*α*	Shear stress reduction ratio	DV
Specimen condition	*V_f_*	Fiber volume fraction	IV
	θ	Biaxial angle	IV

^1^ Constituents properties from the WWFE-III [26]. ^2^ Assumed properties.

**Table 3:** Design variables of each characterization sequence.

Step Number	Implicit Function (*f*(*x*))	*n*	DVs (*x*)
Step 1	$f_{E_{\mathrm{UDL}}}(E_{f1},V_{f})$	1	*E_f_*_1_
Step 2	$f_{E_{\mathrm{BX}}}(E_{f1},E_{m},V_{f},\theta )$	1	*E_m_*
Step 3	$f_{X_{\mathrm{UDL}}}(E_{f1},E_{m},T_{f},V_{f})$ $\;f_{N_{\mathrm{UDL}}}\left(E_{f1},E_{m},T_{f},C_{f},b_{f},m_{f},V_{f},{\tilde{\sigma }}_{max}\right)$	3	*T_f_*, *m_f_*, *b_f_*
Step 4	$f_{X_{f,\mathrm{BX}}}\left(E_{f1},E_{m},T_{m},C_{m},\alpha ,V_{f},\theta \right)$ $\;f_{N_{f,\mathrm{BX}}}\left(E_{f1},E_{m},T_{m},C_{m},b_{m},m_{m},\alpha ,V_{f},\theta ,{\tilde{\sigma }}_{max}\right)$	5	*T_m_*, *C_m_*, *m_f_*, *b_f_*, *α*

**Table 4:** Information on panels fabricated as specimens using a filament winding machine.

Laminates	Lay Up	Volume Fraction
UDL	[0°]*_s_*	60%
BX30	[+30°/−30°]*_s_*	52%
BX60	[+60°/−60°]*_s_*	52%

**Table 5:** Summary of averaged static test result under different temperature (values in parentheses indicate standard deviation).

Properties	Unit	Temperature	UDL	BX30	BX60
Elastic Modulus	GPa	LT	137.9 ^1^	36.8 ^1^	8.3 ^1^
		RT	137.8 ^2^ (3.9)	33.6 ^2^ (0.5)	7.6 ^2^ (0.2)
		HT	137.1 ^1^	26.7 ^1^	4.8 ^1^
Strength	MPa	LT	2566.0 (6.8)	213.7 (2.1)	35.3 (0.6)
		RT	2644.6 (24.3)	233.6 (2.3)	39.1 (1.9)
		HT	2477.5 (11.7)	144.4 (3.1)	23.0 (0.9)

^1^ Estimated elastic moduli of UDL, BX30, and BX60 at LT and HT were calculated based on the OFM and CLT. ^2^ Elastic moduli of UDL, BX30, and BX60 at RT measured on 0.05–0.25% of strain.

**Table 6:** Constituent material properties, determined from the UDL, BX30 and BX60 test results.

Group	Properties	Unit	LT	RT	RT (TDS)	HT	*a_p_*	*b_p_*	*c_p_*
Carbon Fiber (T700)	*E_f_*_1_	GPa	228.46	227.37	230	226.39	+3.49 × 10^−6^	−1.65 × 10^−2^	227.37
	*T_f_*	MPa	4251.5	4367.8	4900	4104.6	−4.94 × 10^−2^	−1.42	4367.8
	*b_f_*	MPa	10,525.4	9304.0	-	6280.5	−2.53 × 10^−1^	−3.52 × 10^1^	9304.0
	*m_f_*	-	6.77	7.66	-	8.1	−5.75 × 10^−5^	+9.95 × 10^−3^	7.7
Epoxy resin (Recyclamine^®^)	*E_m_*	GPa	4.03	3.11	2.84	2.03	−3.08 × 10^−5^	−1.62 × 10^−2^	3.11
	*T_m_*	MPa	37.6	47.7	72.5	25.0	−4.26 × 10^−3^	−1.22 × 10^−1^	47.7
	*C_m_*	MPa	58.5	81.0	-	35.1	−8.90 × 10^−3^	−2.32 × 10^−1^	81.0
	*b_m_*	MPa	107.1	117.2	-	108.0	−2.48 × 10^−3^	−5.11 × 10^−3^	117.2
	*m_m_*	-	5.9	6.7	-	4.9	−3.41 × 10^−4^	−1.04 × 10^−2^	6.7
Nonlinearity of matrix	*α*	-	0.48	0.64	-	0.37	−5.57 × 10^−5^	−1.16 × 10^−3^	0.64

## Data Availability

Data are contained within the article.

## Appendix A. Review of a Micro-Mechanics of Fatigue (MMFatigue)

The MMFatigue is a micromechanics-based fatigue method to predict the number of cycles to failure of composites under cyclic loads, as developed as an extension of the micro-mechanics of failure (MMF) [18,19]. In this approach, the fatigue behavior of composite materials under cyclic loads is analyzed by decomposing the applied fatigue loads into two main components: mean stress ($\sigma_{\mathrm{mean}}^{\left(i\right)}$) and amplitude stress ($\sigma_{\mathrm{amp}}^{\left(i\right)}$), which are calculated in the micromechanical models. These are used to compute the equivalent stress under fatigue conditions.

An equivalent stress is defined as representing the six component multi-axial stresses, simplifying the analysis of fatigue life under cyclic loading; however, with a condition that all the stresses are all in the same phase and same frequence. The amplitude equivalent stress of the matrix is defined as follows:

(A1) $$\sigma_{\mathrm{eq}}^{\mathrm{amp}}=\frac{\left(\beta -1\right)I_{1}^{\mathrm{amp}}+\sqrt{{\left(\beta -1\right)}^{2}{\left(I_{1}^{\mathrm{amp}}\right)}^{2}+4\beta {\left(\sigma_{\mathrm{VM}}^{\mathrm{amp}}\right)}^{2}}}{2\beta }$$

where $\beta =C_{m}/T_{m}$. The $I_{1}^{\mathrm{amp}}$, $I_{2}^{\mathrm{amp}}$, and $\sigma_{\mathrm{vm}}^{\mathrm{amp}}$ are the invariants derived from the micro-stress tensor $\sigma_{ij}$ of the matrix in RVEs:

(A2) $$I_{1}^{\mathrm{amp}}=\sigma_{ii}^{\mathrm{amp}},\;I_{2}^{\mathrm{amp}}=\frac{1}{2}\left(\sigma_{ii}^{\mathrm{amp}}\sigma_{jj}^{\mathrm{amp}}-\sigma_{ij}^{\mathrm{amp}}\sigma_{ji}^{\mathrm{amp}}\right)$$

(A3) $$\sigma_{\mathrm{VM}}^{\mathrm{amp}}=\sqrt{{\left(I_{1}^{\mathrm{amp}}\right)}^{2}-3I_{2}^{\mathrm{amp}}}$$

The mean equivalent stress is also defined in the same manner as in Equations (A2) and (A3), e.g., the superscript “amp” replaced with the “mean”.

Fatigue life is calculated using a CLD based on the mean and amplitude of the equivalent stress. The modified Goodman model (Figure A1a) is applied to relate the CLD to both tension and compression strength, resulting in an effective stress model. This approach enables comparison with the SN curve regardless of the stress ratio. Fatigue loads are divided into mean and amplitude components, which are used to calculate effective stress, and this value is then compared to the SN curve to estimate fatigue life.

The effective stress is defined to consider the effect of the mean stress on the SN curve:

(A4) $$\sigma_{\mathrm{eff}}=\frac{\sigma_{\mathrm{eq}}^{\mathrm{amp}}T}{\frac{T+C}{2}-\left|\sigma_{\mathrm{eq}}^{\mathrm{mean}}-\frac{T-C}{2}\right|}$$

where *T* represents the tensile strength of constituents and *C* denotes compressive strength.

The SN curve is divided into three regions as shown in Figure A1b from Talreja’s research [8]. In the high-stress region (A: Strength), immediate fiber breakage and resin cracking occur, resembling static tensile failure. In the progressive fatigue damage region (B: Fatigue Life), resin cracking leads to fiber–matrix debonding and fiber breakage, ultimately causing fatigue failure. In the fatigue limit region (C: Stress Limit), minor resin cracks form at low stress levels but do not propagate, allowing the material to achieve infinite fatigue life.

In this method, Basquin’s model [25], which plots both the *x*- and *y*-axes on a logarithmic scale, is used to represent the SN curve in terms of the effective stress:

(A5) $$\sigma_{\mathrm{eff}}=b{N_{f}}^{-\frac{1}{m}}$$

where *N_f_* is the number of cycles to failure, *b* the y-intercept of the logarithmic SN curve, and *m* the inverse of the slope.

The fatigue models of amplitude equivalent, mean equivalent, and effective stress for the fiber is assumed to take the same form as the matrix (A1–A5). However, one stress component along the fiber direction is considered instead of all six components of stresses.
